# Supplementary material for: Heat adaptation of phage T7 under an extended genetic code
Source: Virus Evol. 2021 Dec 1;7(2):veab100. doi: 10.1093/ve/veab100 (PMC8923235; doi:10.1093/ve/veab100)
Supplement: veab100_Supp [file veab100_supp.zip › Sequencing_Analysis.zip!/Sequencing_Analysis/S3/evidence/JC_SIDE_2_48.html]

BRESEQ :: Evidence


| New junction evidence | | | | | | | | | | | |
| --- | --- | --- | --- | --- | --- | --- | --- | --- | --- | --- | --- |
|  | | seq id | position | reads (cov) | reads (cov) | score | skew | freq | annotation | gene | product |
| \* | ? | NC\_001604.1 | = 8335 | 1786 (1.040) | 7 (0.000) | 6/584 | 44.4 | 0.39% | intergenic (–/–) | *–/–* | –/– |
|  | ? | NC\_001604.1 | 8939 = | 1866 (1.110) |  |  |  |  | intergenic (–/–) | *–/–* | –/– |
| Rejected: Coverage evenness skew score above cutoff. | | | | | | | | | | | |
| Rejected: Frequency below/above cutoff threshold. | | | | | | | | | | | |

Only 100 of 1906 total aligned reads displayed.  

`AAGTACAGACTACGTGCTGGTAAGAAGTCAGAGTTAGCGTACTTAGAGAAAGACCTAGCGAAAGCAGACTTCTATAAAGAACTCTTTGAGAAACATAAGGATAAATGTTATGCATAACTTCAAGTCAA‑CCCCACCTGCCGACAGCCTATCTGATGACTTCACATCTTGCTCAGAGT‑GGTGCCGAAAGATGTGGGAAGAGACATTCGACGATGCGTACATCAAGCTGTATGAACTTTGGAAATCGAGAGGTCAATGACTATGTCAAACGTAAATACAGGTTCACTTAGTGTGGACAATAAGAAGTTTTGGGCTACCGTAGAGTCCTCGGAGCATTCCTTCGAGGTTCCAATCTACGCTGAGACCCTAGACGAAGCTCTGGAGTTAGCCGAATGGCAATACGTTCCGGCTGGCTTTGAGGTTACTCGTGTGCGTCCTTGTGTAGCACCGAAGTAATACGACTCACTATTAGGGAAGACTCCCTCTGAGAAACCAAACGAAACCTAAAGGAGATTAACATTATGGCTAAGAAGATTTTCACCTCTGCGCTGGGTACCGCTGAACCTTACGCTTACATCGCCAAGCCGGACTACGGCAACGAAG  >  NC_001604.1/8640‑9239`  

                                                                                                                                                                                                                                                                                                             `|`                                                                                                                                                                                                                                                                                                              
  `gTACAGACTACGTGCTGGTAAGAAGTCAGAGTTAGCGTACTTAGAGAAAGACCTAGCGAAAGCAGACTTCTATAAAGAACTCTTTGAGAAACATAAGGATAAATGTTATGCATAACTTCAAGTCAA‑CCCCACCTGCCGACAGCCTATCTGATGACTTCACATCTTGCTCAGAGT‑GGTGCCGAAAGATGTGGGAAGAGACATTCGACGATGCGTACATCAAGCTGTATGAACTTTGGAAATCGAGAGGTCAATGACTATGTCAAACGTAAATACAGGTTCACTTAGTGTGGACAATaagaag                                                                                                                                                                                                                                                                                                           <  1:205258/301‑1 (MQ=255)`  
   `tACAGACTACGTGCTGGTAAGAAGTCAGAGTTAGCGTACTTAGAGAAAGACCTAGCGAAAGTAGACTTCTATAAAGAACTCTTTGAGAAACATAAGGATAAATGTTATGCATAACTTCAAGTCAA‑CCCCACCTGCCGACAGTCTATCTGATGACTTCACATCTTGCTCAGAGT‑GGTGCCGAAAGATGTGGGAATAGACATTCGACGATGCGTACATCAAGCTGTATGAACTTTGGAAATCTAGAGGTCAATGACTATGTCAAACGTAAATACAGGTTCACTTAGTGTGGACAATAAGAAGt                                                                                                                                                                                                                                                                                                          <  1:343143/301‑1 (MQ=255)`  
    `aCAGACTACGTGCTGGTAAGAAGTCAGAGTTAGCGTACTTAGAGAAAGACCTAGCGAAAGCAGACTTCTATAAAGAACTCTTTGAGAAACATAAGGATAAATGTTATGCATAACTTCAAGTCAA‑CCCCACCTGCCGACAGCCTATCTGATGACTTCACATATTGCTCAGAGT‑GGTGCCAAAAGATGTGGGAAGAGACATTCGACGATGCGTACATCAAGCTGTATGAACTTTGGAAATCGAGAGGTCAATGACTATGTCAAACGTAAATACAGGTTCACTTAGTGTGGACAATAAGAAGtt                                                                                                                                                                                                                                                                                                         >  1:263093/1‑301 (MQ=255)`  
       `gACTACGTGCTGGTAAGAAGTCAGAGTTAGCGTACTTAGAGAAAGACCTAGCGAAAGCAGACTTCTATAAAGAACTCTTTGAGAAACATATGGATAAATGTTATGCATAACTTCAAGTCAA‑CCCCACCTGCCGACAGCCTATCTGATGACTTCACATCTTGCTCAGAGT‑GGTGCCGAAAGATGTGGGAAGAGACATTCGACGATGCGTACATCAAGCTGTATGAACTTTGGAAATCGAGAGGTCAATGACTATGTCAAACGTAAATACAGGTTCACTTAGTGTGGACAATaagaactgtcc                                                                                                                                                                                                                                                                                                      >  1:104569/1‑295 (MQ=255)`  
        `aCTACGTGCTGGTAAGAAGTCAGAGTTATCGTACTTAGAGAAAGACCTAGCGAAAGCAGACTTCTATAAAGAACTCTTTGAGAAACATAAGGATAAATGTTATGCATAACTTCAAGTCAACCCCCACCTGCCGACAGCCTATCTGATGACTTCACATCTTGCTCAGAGT‑GGTGCCGAAAGATGTGGGAAGAGACATTCGACGATGCGTACATCAAGCTGTATGAACTTTGGAAATCGAGAGGTCAATGACTATGTCAAACGTAAATACAGGTTCACTTAGTGTGGACAATAAGAATTTTTg                                                                                                                                                                                                                                                                                                      >  1:183779/1‑301 (MQ=255)`  
                  `gggctcggagatgtgtataagagacaggagaAAGACCTAGCGAAAGCAGACTTCTATAAAGAACTCTTTGAGAAACATAAGGATAAATGTTATGCATAACTTCAAGTCAA‑CCCCACCTGCCGACAGCCTATCTGATGACTTCACATCTTGCTCAGAGT‑GGTGCCGAAAGATGTGGGAAGAGACATTCGACGATGCGTACATCAAGCTGTATGAACTTTGGAAATCGAGAGGTCAATGACTATGTCAAACGTAAATACAGGTTCACTTAGTGTGGACAATAAGAAGTTTTGGGCTACCGTag                                                                                                                                                                                                                                                                                           <  1:11752/274‑1 (MQ=255)`  
                  `ggTAAGAAGTCAGAGTTAGCGTACTTAGAGAAAGACCTAGCGAAAGCAGACTTCTATAAAGAACTCTTTGAGAAACATAAGGATAAATGTTATGCATAACTTCAAGTCAA‑CCCCACCTGCCGACAGCCTATCTGATGACTTCACATCTTGCTCAGAGT‑GGTGCCGAAAGATGTGGGAAGAGACATTCGACGATGCGTACATCAAGCTGTATGAACTTTGGAAATCGAGAGGTCAATGACTATGTCAAACGTAAATACAGGTTCACTTAGTGTGGACAATAAGAAGTTTTGGGCTACCGTaa                                                                                                                                                                                                                                                                                           >  1:245050/1‑300 (MQ=255)`  
                  `ggTAAGAAGTCAGAGTTAGCGTACTTAGAGAAAGACCTAGCGAAAGCAGACTTCTATAAAGAACTCTTTGAGAAACATAAGGATAAATGTTATGCATAACTTCAAGTCAA‑CCCCACCTGCCGACAGCCTATCTGATGACTTCACATCTTGCTCAGAGT‑GGTGCCGAAAGATGTGGGAAGAGACATTCGACGATGCGTACATCAAGCTGTATGAACTTTGGAAATCGAGAGGTCAATGACTATGTCAAACGTAAATACAGGTTCACTTAGTGTGGACAATAAGAAGTTTTGGACTACCGTag                                                                                                                                                                                                                                                                                           >  1:42539/1‑301 (MQ=255)`  
                      `agaagTCAGAGTTAGCTTACTTAGAGAAAGACATAGCGAAAGCAGACTTCTATAAAGAACTCTGTGAGAAACATAAGGATAAATGTTATGCATAACTTCAAGTCAA‑CCCCACCTGCCGACAGCCTATCTGATGACTTCACATCTTGCTCAGAGT‑GGTGCCGAAAGATGTGGGAAGAAACATTCGACGATGCGTACATCAAGCTGTATGAACTTTGGAAATCGAGAGGTCAATGACTATGTCAAACGTAAATACAGGTTCACTTAGTGTGGACAATAAGAAGTTTTGGGCTACCGTAGAGTc                                                                                                                                                                                                                                                                                       <  1:324529/301‑1 (MQ=255)`  
                       `aaagTCAGAGTTAGCGTACTTAGAGAAAGACCTAGCGAAAGCAGACTTCTATAAAGAACTCTTTGAGAAACATAAGGATAAATGTTATGCATAACTTCAAGTCAA‑CCCCACCTGCCGACAGCCTATCTGATGACTTCACATCTTGCTCAGAGT‑GGTGCCGAAAGATGTGGGAAGAGACACTCGACGATGCGTACATCAAGCTGTATGAACTTTGGAAATCGAGAGGTCAATGACTATGTCAAACGTAAATACAGGTTCACTTAGTGTGGACAATAAGAAGTTTTGGGCTACCGTAGAGTcc                                                                                                                                                                                                                                                                                      <  1:302449/300‑1 (MQ=255)`  
                          `gTCAGAGTTAGCGTACTTAGAGAAAGACCTAGCGAAAGCAGACTTCTATAAAGAACTCTTTGAGAAACATAAGGATAAATGTTATGCATAACTTCAAGTCAA‑CCCCACCTGCCGACAGCCTATCTGATGACTTCACATCTTGCTCAGAGT‑GGTGCCGAAAGATGTGGGAAGAGACATTCGACGATGCGTACATCAAGCTGTATGAACTTTGGAAATCGAGAGGTCAATGACTATGTCAAACGTAAATACAGGTTCACTTAGTGTGGACAATAAGAAGTTTTGGGCTACCGTAGAGTCCTCg                                                                                                                                                                                                                                                                                   >  1:297291/1‑301 (MQ=255)`  
                           `tCAGAGTTAGCGTACTTAGAGAAAGACCTAGCGAAAGCAGACTTCTATAAAGAACTCTTTGAGAAACATAAGGATAAATGTTATGCATAACTTCAAGTCAA‑CCCCACCTGCCGACAGCCTATCTGATGACTTCACATCTTGCTCAGAGT‑GGTGCCGAAAGATGTGGGAAGAGACATTCGACGATGCGTACATCAAGCTGTATGAACTTTGGAAATCGAGAGGTCAATGACTATGTCAAACGTAAATACAGGTTCACTTAGTGTGGACAATAAGAAGTTTTGGGCTACCGTAGAGTCCTCgg                                                                                                                                                                                                                                                                                  <  1:207252/301‑1 (MQ=255)`  
                            `aagagTTAGCGTACTTAGAGAAAGACCTAGCGAAAGCAGACTTCTATAAAGAACGCTTTGAGAAACATAAGGATAAATGTTATGCATAACTTCAAGTCAA‑CCCCACCTGCCGACAGCCTATCTGATGACTTCACATCGTGCTCAGAGT‑GGTGCCGAAAGATGTGGGAAGAGACATTCGACGATGCGTACATCAAGCTGTATGAACTTTGGAAATCGAGAGGTCAATGACTATGTAAAACGTAAATACAGGTTCACTTAGTGTGGACAATAAGAAGTTTTGGGCTACCGTAGAGTCCTCGGa                                                                                                                                                                                                                                                                                 <  1:272939/300‑1 (MQ=255)`  
                                   `agagTACTTAGAGAAAGACCTAGCGAAAGCAGACTTCTATAAAGAACTCTTTGAGAAACATAAGGATAAATGTTATGCATAACTTCAAGTCAA‑CCCCACCTGCCGACAGACTATCTGATGACTTCACATCTTGATCAGAGT‑GGTGCCGAAAGATGTGGGAAGAGACATTCGACGATGCGTACATCAAGCTGTATGAACTTTGGAAATCGAGAGGTCAATGACTATGTCAAACGTAAATACAGGTTCACTTAGTGTGGACAATAAGAAGTTTTGGGCTACCGTAGAGTCCTCGGAGCATTcc                                                                                                                                                                                                                                                                          <  1:154624/298‑1 (MQ=255)`  
                                   `aGCGTACTTAGAGAAAGACCTAGCGAAAGCAGACTTCTATAAAGAACTCTTTGAGAAACATAAGGATAAATGTTATGCATAACTTCAAGTCAA‑CCCCACCTGCCGACAGCCTATCTGATGACTTCACATCTTGCTCAGAGT‑GGTGCCGAAAGATGTGGGAAGAGACATTCGACGATGCGTACATCAAGCTGTATGAACTTTGGAAATCGAGAGGTCAATGACTATGTCAAACGTAAATACAGGTTCACTTAGTGTGGACAATAAGAAGTTTTGGGCTACCGTAGAGTCCTCGGAGCATTcc                                                                                                                                                                                                                                                                          <  1:281078/301‑1 (MQ=255)`  
                                       `tACTTAGAGAAAGACCTAGCGAAAGCAGACTTCTATAAAGAACTCTTTGAGAAACATAAGGATAAATGTTATGCATAACTTCAAGTCAA‑CCCCACCTGCCGACAGCCTATCTGATGACTTCACATCTTGCTCAGAGT‑AGTGCCGAAAGATGTGGGAAGAGACATTCGACGATGCGTACATCAAGCTGTATGAACTTTGGAAATCGAGAGGTCAATGACTATGTCAAACGTAAATACAGGTTCACTTAGTGTGGACAATAAGAAGTTTTGGGCTACCGTAGAGTCCTCGGAGCATTCCTTCg                                                                                                                                                                                                                                                                      >  1:143348/1‑301 (MQ=255)`  
                                                   `gACCTAGCGAAAGCAGACTTCTATAAAGAACTCTTTGAGAAACATAAGGATAAATGTTATGCATAACTTCAAGTCAA‑CCCCACCTGCCGACAGCCTATCTGATGACTTCACATCTTGCTCAGAGT‑GGTGCCGAAAGATGTGGGAAGAGACATTCGACGATGCGTACATCAAGCTGTATGAACTTTGGAAATCGAGAGGTCAATGACTATGTCAAACGTAAATACAGGTTCACTTAGTGTGGACAATAAGAAGTTTTGGGCTACCGTAGAGTCCTCGGAGCATTCCTTCGAGGTTCCAATCt                                                                                                                                                                                                                                                          >  1:146282/1‑301 (MQ=255)`  
                                                       `gtataagagacagcTTCTATAAAGAACTCTTTGAGAAACATAAGGATAAATGTTATGCATAACTTCAAGTCAA‑CCCCACCTGCCGACAGCCTATCTGATGACTTCACATCTTGCTCAGAGT‑GGTGCCGAAAGATGTGGGAAGAGACATTCGACGATGCGTACATCAAGCTGTATGAACTTTGGAAATCGAGAGGTCAATGACTATGTCAAACGTAAATACAGGTTCACTTAGTGTGGACAATAAGAAGTTTTGGGCTACCGTAGAGTCCTCGGAGCATTCCTTCGAGGTTCCAATCTACGc                                                                                                                                                                                                                                                      <  1:81840/288‑1 (MQ=255)`  
                                                       `gacagAAAGCAGACTTCTATAAAGAACTCTTTGAGAAACATAAGGATAAATGTTATGCATAACTTCAAGTCAA‑CCCCACCTGCCGACAGCCTATCTGATGACTTCACATCTTGCTCAGAGT‑GGTGCCGAAAGATGTGGGAAGAGACATTCGACGATGCGTACATCAAGCTGTATGAACTTTGGAAATCGAGAGGTCAATGACTATGTCAAACGTAAATACAGGTTCACTTAGTGTGGACAATAAGAAGTTTTGGGCTACCGTAGAGTCCTCGGAGCATTCCTTCGAGGTTCCAATCTACGc                                                                                                                                                                                                                                                      <  1:107299/297‑1 (MQ=255)`  
                                                        `aGCGAAAGCAGACTTCTATAAAGAACTCTTTGAGAAACATAAGGATAAATGTTATGCATAACTTCAAGTCAA‑CCCCACCTGCCGACAGACTATCTGATGACTTCACATCTTGCTCAGAGT‑GGTGCCGAAAGATGTGGGAAGAGACATTCGACGATGCGTACATCAAGCTGTATGAACTTTGGAAATCGAGAGGTCAATGACTATGTCAAACGTAAATACAGGTTCACTTAGTGTGGACAATAAGAAGTTTTGGGCTACCGTAGAGTCCTCGGAGCATTCCTTCGAGGTTCCAATCTACGCt                                                                                                                                                                                                                                                     <  1:97589/301‑1 (MQ=255)`  
                                                            `aaaGCAGACTTCTATAAAGAACTCTTTGAGAAACATAAGGATAAATGTTATGCATAACTTCAAGTCAA‑CCCCACCTGCCGACAGCCTATCTGATGACTTCACATCTTGCTCAGAGT‑GGTGCCGAAAGATGTGGGAAGAGACATTCGACGATGCGTACATCAAGCTGTATGAACTTTGGAAATCGAGAGGTCAATGACTATGTCAAACGTAAATACAGGTTCACTTAGTGTGGACAATAAGAAGTTTTGGGCTACCGTAGAGTCCTCGGAGCATTCCTTCGAGGTTCCAATCTACGCTgaga                                                                                                                                                                                                                                                 <  1:84269/301‑1 (MQ=255)`  
                                                                    `cgtgggctcggagatgtgtataagagacagggATAAATGTTATGCATAACTTCAAGTCAA‑CCCCACCTGCCGACAGCCTATCTGATGACTACACATCTTGCTCAGAGT‑GGTGCCGAAAGATGTGGGAAGAGACATTCGACGATGCGTACATCAAGCTGTATGAACTTTGGAAGTCGAGAGGTCAATGACTATGTCAAACGTAAATACAGGTTCACTTAGTGTGGACAATAAGAAGTTTGGGGCTACCGTAGAGTCCTCGGAGCATTCCTTCGAGGTTCCAATCTACGCTGAGACCCTAGAc                                                                                                                                                                                                                                         <  1:5011/271‑1 (MQ=255)`  
                                                                    `cTTCTATAAAGAACTCTTTGAGAAACATAAGGATAAATGTTATGCATACCTTCAAGTCAA‑CCCCACCTGCCGACAGCCTATCTGATGACTTCACATCTTGCTCAGAGT‑GGTGCCGAAAGATGTGGGAAGAGACATTCGACGATGCGTACATCAAGCTGTATGAACTTTGGAAATCGAGAGGTCAATGACTATGTCAAACGTAAATACAGGTTCACTTAGTGTGGACAATAAGAAGTTTTGGGCTACCGTAGAGTCCTCGGAGCATTCCTTCGAGGTTCCAATCTACGCTGAGACCCTAGAc                                                                                                                                                                                                                                         <  1:236353/301‑1 (MQ=255)`  
                                                                    `cTTCTATAAAGAACTCTTTGAGAAACATAAGGATAAATGTTATGCATAACTTCAAGTCAA‑CCCCACCTGCCGACAGCCTATCTGATGACTTCACATCTTGCTCAGAGT‑GGTGCCGAAAGATGTGGGAAGAGACATTCGACGATGCGTACATCAAGCTGTATGAACTTTGGAAATCGAGAGGTCAATGACTATGTCAAACGTAAATACAGGTTCACTTAGTGTGGACAATAAGAAGTTTTGGGCTACCGTAGAGTCCTCGGAGCATTCCTTCGAGGTTCCAATCTACGCTGAGACCCTAGAc                                                                                                                                                                                                                                         >  1:164512/1‑301 (MQ=255)`  
                                                                      `tCTATAAAGAACTCTTTTAGAAACATAAGGATAAATGTTATGCATAACTTCAAGTCAA‑CCCCACCTGCCGACAGCCTATCTGATGACTTCACATCTTGCTCAGAGT‑GGTGCCGAAAGATGTGGGAAGAGACATTCGACGATGCGTACATCAAGCTGTATGAACTTTGGAAATCGCGAGGTCAATGACTATGTCAAACGTAAATACAGGTTCACTTAGTGTGGACAATAAGAAGTTTTGGGCTACCGTAGAGTCCTCGGAGCATTCCTTCGAGGTTCCAATCTACGCTGCGACCATAGACGa                                                                                                                                                                                                                                       >  1:94454/1‑301 (MQ=255)`  
                                                                      `tCTATAAAGAACTCTTTGAGAAACATAAGGATAAATGTTATGCATAACTTCAAGTCAA‑CCCCACCTGCCGACAGCCTATCTGATGACTTCACATCTTGCTCAGAGT‑GGTGCCGAAAGATGTGGGAAGAGACATTCGACGATGCGTACATCAAGCTGTATGAACTTTGGAAATCGAGAGGTCAATGACTATGTCAAACGTAAATACAGGTTCACTTAGTGTGGACAATAAGAAGTTTTGGGCTACCGTAGAGTCCTCGGAGCATTCCTTCGAGGTTCCAATCTACGCTGAGACCCTAGACgc                                                                                                                                                                                                                                       >  1:283935/1‑300 (MQ=255)`  
                                                                      `tCTATAAAGAACTCTTTGAGAAACATAAGGATAAATGTTATGCATAACTTCAAGTCAA‑CCCCACCTGCCGACAGCCTATCTGATGACTTCACATCTTGCTCAGAGT‑GGTGCCGAAAGATGTGGGAAGAGACATTCGACGATGCGTACATCAAGCTGTATGAACTTTGGAAATCGAGAGGTCAATGACTATGTCAAACGTAAATACAGGTTCACCTAGTGTGGACAATAAGAAGTTTTGGGCTACCGTAGAGTCCTCGGAGCATTCCTTCGAGGTTCCAATCTACGCTGAGACCCTAGACGa                                                                                                                                                                                                                                       >  1:310346/1‑301 (MQ=255)`  
                                                                                     `ttGAGAAACATAAGGATAAATGTTATGCATAACTTCAAGTCAA‑CCCCACCTGCCGACAGCCTATCTGATGACTTCACATCTTTCTCAGAGT‑GGTGCCGAAAGATGTGGGAAGAGACATTCGACGATGCGTACATCAAGCTGTATGAACTTTGGAAATCGAGAGGTCAATGACTATGTCAAACGTAAATACAGGTTCACTTAGTGTGGACAATAAGAAGTTTTGGGCTACCGTAGAGTCCTCGGAGCATTCCTTCGAGGTTCCAATCTACGCTGAGACCCTAGACGAAGCTCTGGAGTTAGc                                                                                                                                                                                                                        <  1:62366/301‑1 (MQ=255)`  
                                                                                            `aCATAAGGATAAATGTTATGCATAACTTCAAGTCAA‑CCCCACCTGCCGACAGCCTATCTGATGACTTCACATCTTGCTCAGAGT‑GGTGCCGAAAGATGTGGGAAGAGACATTCGACGATGCGTACATCAAGCTGTATGAACTTTGGAAATCGAGAGGTCAATGACTATGTCAAACGTAAATACAGGTTCACTTAGTGTGGACAATAAGAAGTTTTGGGCTACCGTAGAGTCCTCGGAGCATTCCTTCGAGGTTCCAATCTACGCTGAGACCCTAGACGAAGCTCTGGAGTTAGCCGAATgg                                                                                                                                                                                                                 >  1:202044/1‑301 (MQ=255)`  
                                                                                             `cATAAGGATAAATGTTATGCATAACTTCAAGTCAA‑CCCCACCTGCCGACAGCCTATCTGATGACTTCACATCTTGCTCAGAGT‑GGTGCCGAAAGATGTGGGAAGAGACATTCCACGATGCGTACATCAAGCTGTATGAACTTTGGAAATCGAGAGGTCAATGACTATGTCAAACGTAAATACAGGTTCACTTAGTGTGGACAATAAGAAGTTTTGGGCTACCGTAGAGTCCTCGGAGCATTCCTTCGAGGTTCCAATCTACGCTGAGACCCTAGACGAAGCTCTGGATTTAGCCGAATGGc                                                                                                                                                                                                                >  1:71516/1‑301 (MQ=255)`  
                                                                                                  `ggATAAATGTTATGCATAACTTCAAGTCAA‑CCCCACCTGCCGACAGCCTATCTGATGACTTCACATCTTGCTCAGAGT‑GGTGCCGAAAGATGTGGGAAGAGACATTCGACGATGCGTACATCAAGCTGTATGAACTTTGGAAATCGAGAGGTCAATGACTATGTCAAACGTAAATACAGGTTCACTTAGTGTGGACAATAAGAAGTTTTGGGCTACCGTAGAGTCCTCGGAGCATTCCTTCGAGGTTCCAATCTACGCTGAGACCCTAGACGAAGCTCTGGAgctgtctcttatacacatc                                                                                                                                                                                                           >  1:171799/1‑283 (MQ=255)`  
                                                                                                  `ggATAAATGTTATGCATAACTTCAAGTCAA‑CCCCACCTGCCGACAGCCTATCTGATGACTTCACATCTTGCTCAGAGT‑GGTGCCGAAAGATGTGGGAAGAGACATTCGACGATGCGTACATCAAGCTGTATGAACTTTGGAAATCGAGAGGTCAATGACTATGTCAAACGTAAATACAGGTTCACTTAGTGTGGACAATAAGAAGTTTTGGGCTACCGTAGAGTCCTCGGAGCATTCCTTCGAGGTTCCAATCTACGCTGAGACCCTAGACGAAGCTCTGGAGTTAGCCGAATGGCAATAc                                                                                                                                                                                                           <  1:69009/301‑1 (MQ=255)`  
                                                                                                  `ggATAAATGTTATGCATAACTTCAAGTCAA‑CCCCACCTGCCGACAGCCTATCTGATGACTTCACATCTTGCTCAGACT‑GGTGCCGAAAGATGTGGGAAGAGACATTCGACGATGCGTACATCAAGCTGTATGAACTTTGGAAATCGAGAGGTCAATGACTATGTCAAACGTAAATACAGGTTCACTTAGTGTGGACAATAAGAAGTTTTGGGCTACCGTAGAGTCCTCGTAGCATTCCTTCGAGGTTCCACTCTACGCTGAGACCCTAGACGAAGCTCTGGAGTTAGCCGAATGGCAATAc                                                                                                                                                                                                           >  1:316710/1‑301 (MQ=255)`  
                                                                                                    `acagaTGTAATGCATAACTTCAAGTCAA‑CCCCACCTGCCGACAGCCTATCTGATGACTTCACATCTTGCTCAGAGT‑GGTGCCGAAAGATGTGGGAAGAGACATTCGACGATGCGTACATCAAGCTGTATGAACTTTGGAAATCGAGAGGTCAATGACTATGTCAAACGTAAATACAGGTTCACTTAGTGTGGACAATAAGAAGTTTTGGGCTACCGTAGAGTCCTCGGAGCATTCCTTCGAGGTTCCAATCTACGCTGAGACCCTAGACGAAGCTCTGGAGTTAGCCGAATGGCAATACGt                                                                                                                                                                                                         <  1:306374/297‑1 (MQ=255)`  
                                                                                                       `aaTGTTATGCATAACTTCAAGTCAA‑CCCCACCTGCCGACAGCCTATCTGATGACTTCACATCTTGCTCAGAGT‑GGTGCCGAAAGATGTGGGAAGAGACATTCGACGATGCGTACATCAAGCTGTATGAACTTTGGAAATCGAGAGGTCAATGACTATGTCAAACGTAAATACAGGTTCACTTAGTGTGGACAATAAGAAGTTTTGGGCTACCGTAGAGTCCTCGGAGCATTCCTTCGAGGTTCCAATCTACGCTGAGACCCTAGACGAAGCTCTGGAGTTAGCCGAATGGCAATACGTTcc                                                                                                                                                                                                      <  1:224065/301‑1 (MQ=255)`  
                                                                                                        `aTGTTATGCATAACTTCAAGTCAA‑CCCCACCTGCCGACAGCCTATCTGATGACTTCACATCTTGCTCAGAGT‑GGTGCCGAAAGATGTGGGAAGAGACATTCGACGATGCGTACATCAAGCTGTATGAACTTTGGAAATCTAGAGGTCAATGACTATGTCAAACGTAAATACAGGTTCACTTAGTGTGGACAATAAGAAGTTTTGGGCTACCGTAGAGTCCTCGGAGCATTCCTTCGAGGTTCCAATCTACGCTGAGACCCTAGACGAAGCTCTGGAGTTAGCCGAATGGctgtctcttatt                                                                                                                                                                                                     >  1:294934/1‑290 (MQ=255)`  
                                                                                                               `gCATAACTTCAAGTCAA‑CCCCACCTGCCGACAGCCTATCTGATGACTTCACATCTTGCTCAGAGT‑GGTGCCGAAAGATGTGGGAAGAGACATTCGACGATGCGTACATCAAGCTGTATGAACTTTGGAAATCGAGAGGTCAATGACTATGTCAAACGTAAATACAGGTTCACTTAGTGTGGACAATACGAAGTTTTGGGCTACCGTAGAGTCCTCGGAGCATTCCTTCGAGGTTCCAATCTACGCTGAGACCCTAGACGAAGCTCTGGAGTTAGCCGAATGGCAATACGTTCCggctggct                                                                                                                                                                                              >  1:248300/1‑301 (MQ=255)`  
                                                                                                               `gCATAACTTCAAGTCAA‑CCCCACCTGCCGACAGCCTATCTGATGACTTCACATCTTGCTCAGAGT‑GGTGCCGAAAGATGTGGGAAGAGACATTCGACGATGCGTACATCAAGCTGTATGAACTTTGGAAATCGAGAGGTCAATGACTATGTCAAACGTAAATACAGGTTCACTTAGTGTGGACAATAAGAAGTTTTGGGCTACCGTAGAGTCCTCGGAGCATTCCTTCGAGGTTCCAATCTACGCTGAGACCCTAGACGAAGCTCTGGAGTTAGCCGAATGGCAATACGTTCCggctggct                                                                                                                                                                                              >  1:188700/1‑301 (MQ=255)`  
                                                                                                               `gCATAACTTCAAGTCAA‑CCCCACCTGCCGACAGCCTATCTGATGACTTCACATCTTGCTCAGAGT‑GGTGCCGAAAGATGTGGGAAGAGACATTCGACGATGCGTACATCAAGCTGTATGAACTTTGGAAATCGAGAGGTCAATGACTATGTCAAACGTAAATACAGGTTCACTTAGTGTGGACAATAAGAAGTTTTGGGCTACCGTAGAGTCCTCGGAGCATTCCTTCGAGGTTCCAATCTACGCTGAGACCCTAGACGAAGCTCTGGAGTTAGCCGAATGGCAATACGTTCCggctggct                                                                                                                                                                                              >  1:58497/1‑301 (MQ=255)`  
                                                                                                                     `cTTCAAGTCAA‑CCCCACCTGCCGACAGCCTATCTGATGACTTCACATCTTGCTCAGAGT‑GGTGCCGAAAGATGTGGGAAGAGACATTCGTCGATGCGTACATCAAGCTGTATGAACTTTGGAAATCGAGAGGTCAATGACTATGTCAAACGTAAATACAGGTTCACTTAGTGTGGACAATAAGAAGTTTTGGGCTACCGTAGAGTCCTCGGATCATTCCTTCGAGGTTCCAATCTACGCTGAGACCCTAGACGAAGCTCTGGAGTTAGCCGAATGGCAATACGTTCCGGCTGGCTTTGAgg                                                                                                                                                                                        >  1:174002/1‑301 (MQ=255)`  
                                                                                                                     `cTTCAAGTCAA‑CCCCACCTGCCGACAGCCTATCTGATGACTTCACATCTTGCTCAGAGT‑GGTGCCGAAAGATGTGGGAAGAGACATTCGACGATGCGTACATCAAGCTGTATGAACTTTGGAAATCGAGAGGTCAATGACTATGTCAAACGTAAATACAGGTTCACTTAGTGTGGACAATAAGAAGTTTTGGGCTACCGTAGAGTCCTCGGAGCATTCCTTCGAGGTTCCAATCTACGCTGAGACCCTAGACGAAGCTCTGGAGTTAGCCGAATGGCAATACGTTCCGGCTGGCTTTGAgg                                                                                                                                                                                        >  1:114677/1‑301 (MQ=255)`  
                                                                                                                                 `ccccACCTGCCGACAGCCTATCTGATGACTTCACATCTTGCTCAGAGT‑GGTTCCGAAAGATGTGGGAAGAGACATTCGCCGATGCGTACATCAAGCTGTATGAACTTTGGAAATCGAGAGGTCAATGACTATGTCAAACGTAAATACAGGTTCACTTAGTGTGGACAATAAGAAGTTTTGGGCTACCGTAGAGTCCTCGGAGCATTCCTTCGAGGTTCCAATCTACGCTGAGACCCTAGACGAAGCTCTGGAGTTAGCCGAATGGCAATACGTTCCGGCTGGCTTTGAGGTTACTCGtgtg                                                                                                                                                                             <  1:195954/301‑1 (MQ=255)`  
                                                                                                                                 `ccccACCTGCCGACAGCCTATCTGATGACTTCACATCTTGCTCAGAGT‑GGTGCCGAAAGATGTGGGAAGAGACATTCGACGATGCGTACATCAAGCTGTATGAACTTTGGAAATCGAGAGGTCAATGACTATGTCAAACGTAAATACAGGCTCACTTAGTGTGGACAATAAGAAGTTTTGGGCTACCGTAGAGTCCTCGGAGCATTCCTTCGAGGTTCCAATCTACGCTGAGACCCTAGACGAAGCTCTGGAGTTAGCCGAAGGGCAATACGTTCCGGCTGGCTTTGGGGTTAcgcgatgg                                                                                                                                                                             >  1:220429/1‑297 (MQ=255)`  
                                                                                                                                     `ccctgcccaacggcttacctattgattCACAACCTGGTCAGAATGGGTGCGGAAA‑AAGTGGGAAGGGGCCTTTGACGCTTCGTACATCAAACTGTATTAAATTTTGAAATCCGGAGGTCCAAGGCTATGTCAAACTGAAATACAGGTTCACTTAGTGTGGACAATAAGAAGTTTTGGGCTACCGTAGAGTCCTCGGAGCATTCCTTCGAGGTTCCAATCTACGCTGAGACCCTAGACGAAGCTCTGGAGTTAGCCGAATGGCAATACGTTCCGGCTGGCTTTGAGGTTACTCGTGTGCGTc                                                                                                                                                                         <  1:218242/276‑1 (MQ=255)`  
                                                                                                                                      `ccTGCCGACAGCCTATCTGATGACTTCACATCTTGCTCAGAGT‑GGTGCCGAAAGCTGTGGGAAGAGACATTCGACGATGCGTACATCAAGCTGTATGAACTTTGGAAATCGAGAGGTCAATGACTATGTCAAACGTAAATACAGGTTCACTTAGTGTGGACAATAAGAAGTTTTGGGCTACCGTAGAGTCCTCGGAGCATTCCTTCGAGGTTCCAATCTACGCTGAGACCCTAGACGAAGCTCTGGAGTTAGCCGAATGGCAATACGTTCCGTCTGGCTTTGTGGTTACTCGTGTGCGTcc                                                                                                                                                                        >  1:252750/1‑301 (MQ=255)`  
                                                                                                                                         `gCCGACAGCCTATCTGATGACTTCACATCTTGCTCAGAGT‑GGTGCCGAAAGATGTGGGAAGAGACATTCGACGATGCGTACATCAAGCTGTATGAACTTTGGAAATCGAGAGGTCAATGACTATGTCAAACGTAAATACAGGTTCACTTAGTGTGGACAATAAGAAGTTTTGGGCTACCGTAGAGTCCTCGGAGCATTCCTTCGAGGTTCCAATCTACGCTGAGACCCTAGACGAAGCTCTGGAGTTAGCCGAATGGCAATACGTTCCGGCTGTCTTTGAGGTTATTCGTGTGCGTCCttt                                                                                                                                                                     >  1:92737/1‑300 (MQ=255)`  
                                                                                                                                            `gagacagtagcTGATGACTTCACATCTTACTCAGAGT‑GGTGCCGAAAGATGTGGGAAGAGACATTCGACGATGCGTACATCAAGCTGTATGAACTTTGGAAATCGAGAGGTCAATGACTATGTCAAACGTAAATACAGGTTCACTTAGTGTGGACAATAAGAAGTTTTGGGCTACCGTAGAGTCCTAGGAGCATTCCTTCGAGGTTCCAATCTACGCTGAGACCCTAGACGAAGCTCTGGAGTTAGCCGAATGGCAATACGTTCCGGCTGGCTTTGAGGTTACTCGTGTGCGTCCTTgtgt                                                                                                                                                                  <  1:277062/291‑1 (MQ=255)`  
                                                                                                                                            `gacaagcTAACTGATGACTTCACATCTTGCTCAGAGG‑TGTGCCGAAAGATGTGGGAAGAGACATTCGACGATGCGTACATCAAGCTGTATGAACTTTGGAAATCGAGAGGTCAATGACTATGTCAAACGTAAATACAGGTTCACTTAGTGTGGACAATAAGAAGTTTTAGGCTACCGTAGAGTCCTCGGAGCATTCCTTCGAGGTTCCAATCTACGCTGAAACCCTAGACGAAGCTCTGGAGTTAGCCGAATGGCAATACGTTCCGGCTGGCTTTGAGGTTACTCGTGTGCGTCCTTgtgt                                                                                                                                                                  <  1:44457/295‑1 (MQ=255)`  
                                                                                                                                            `gACAGCCTATCTGATGACTTCACAGCTTGCTCAGAGT‑GGTGCCGAAAGATGTGGGAAGAGACATTCGACGATGCGTACATCAAGCTGTATGAACTTTGGAAATCGAGAGGTCAATGACTATGTCAAACGTAAATACAGGTTCACTTAGTGTGGACAATAAGAAGTTTTGGGCTACCGTAGAGTCCTCGGAGCATTCCTTCGAGGTTCCAATCTACGCTGAGACCCTAGACGAAGCTCTGGAGTTAGCCGAATGGCAATACGTTCCGGCTGGCTTTGAGGTTACTCGTGTGCGTCCTTgtgt                                                                                                                                                                  <  1:177689/301‑1 (MQ=255)`  
                                                                                                                                               `aGCCTATCTGATGACTTCACATCTTGCTCAGAGT‑GGTGCCGAAAGATGTGGGAAGAGACATTCGACGATGCGTACATCAAGCTGTATGAACTTTGGAAATCGAGAGGTCAATGACTATGTCAAACGTAAATACAGGTTCACTTAGTGTGGACAATAAGAAGTTTTGGGCTACCGTAGAGTCCTCGGAGCATTCCTTCGAGGTTCCAATCTACGCTGAGACCCTAGACGAAGCTCTGGAGTTAGCCGAATGGCAATACGTTCCGGCTGGCTTTGAGGTTACTCGTGTGCGTCCTTGTGTAGc                                                                                                                                                               <  1:260958/301‑1 (MQ=255)`  
                                                                                                                                                    `aTCTGATGACTTCGCATCTTGCTCAGAGT‑GGTGCCGAAAGATGTGGGAAGAGACATTCGACGATGCGTACATCAAGCTGTATGAACTTTGGAAATCGAGAGGTCAATGACTATGTCAAACGTAAATACAGGTTCACTTAGTGTGGACAATAAGAAGTTTTGGGCTACCGTAGAGTCCTCGGAGCATTCCTTCGAGGTTCCAATCTACGCTGAGACCCTAGACGAAGCTCTGGAGTTAGCCGAATGGCAATACGTTCCGGCTGGCTTTGAGGTTACTCGTGTGCGTCCTTGTGTAGCACCGa                                                                                                                                                          >  1:214238/1‑301 (MQ=255)`  
                                                                                                                                                           `gatttCACATCTTGCTGAGATT‑GGTGCAGAAAGATGTGGAAAGAGACATTCTACGATGCGTACATCAAGCTGTATGAACTTTGGAAATCGAGAGGTCAATGATTATGTCAAACGTAAATACATGTTCACTTAGTGTGGACAATAAGAAGTTTTGGGCTACCGTAGAGTCCTCGGAGCATTCCTTCGAGGTTCCAATCTACGCTGAGACCCTAGACGAAGCTCTGGAGTTAGCCGAATGGCAATACGTTCCGGCTGGCTTTGAGGTTACTCGTGTGCGTCCTTGTGTAGCACCGAAGTAATa                                                                                                                                                   <  1:78392/298‑1 (MQ=255)`  
                                                                                                                                                           `gaattCACAACTTGCTCAGAGT‑GGTGCCGAAAGATGTTGGAAGAGACATTCGACGATGCGTACATCAAGCTGTATGAACTTTGGAAATCGAGAGGTCAATGACTATGTCAAACGTAAATACAGGTTCACTTAGTGTGGAAAATAAGAAGTTTTGGGCTACCGTAGAGTCCTCGGAGCATTCCTTCGAGGTTCCAATCTACGCTGAGACCCTGGACGAAGCTCTGGAGTTAGCCGAATGGCAATACGTTCCGGCTGGCTTTGAGGTTACTCGTGTGCGCCCTTGTGTAGCACAGAAGTAATa                                                                                                                                                   <  1:227299/298‑1 (MQ=255)`  
                                                                                                                                                            `aagaaagaTCTTGCTCAGAGA‑GGAGCCGAAAGATGTGGGAAGAGACATTCGACGATGCGTACATCAAGCTGTATGAACTTTGGAAATCGAGAGGTCAATGACTATGTCAAACGTAAATACAGGTTCACTTAGTGTGGACAATAAGAAGTTTTGGGCTACCGTAGAGTCCTCGGAGAATTCCTTCGAGGTTCCAATCTACGCTGAGACCCTAGACGAAGCTCTGAAGTTAGCCGAATGGCAATACGTTCCGGCTGGCTTTGAGGTTACCCGTGTGCGTCCTTGTGTAGCACCGAAGTAATAc                                                                                                                                                  <  1:347837/294‑1 (MQ=255)`  
                                                                                                                                                            `aCTTCACATCTTGCTCAGAGT‑GGTGCCGAAAGATGTGGGAAGAGACATTCGACGATGCGTACATCAAGCTGTATGAACTTTGTAAATCGAGAGGTCAATGACTATGTCAAACGTAAATACAGGTTCACTTAGTGTGGACAATAAGAAGTTTTGGGCTACCGTAGAGTCCTCGGAGCATTCCTTCGAGGTTCCAATCTACGCTGAGACCCTAGACGAAGCTCTGGAGTTAGCCGAATGGCAATACGTTCCGGCTGGCTTTGAGGTTACTCGTGTGCGTCCTTGTGTAGCACCGAAGTAATAc                                                                                                                                                  <  1:161512/301‑1 (MQ=255)`  
                                                                                                                                                            `aCTTCACATCTTGCTCAGAGT‑GGTGCCGAAAGATGTGGGAAGAGACATTCGACGATGCGTACATCAAGCTGTATGAACTTTGGAAATCGAGAGGTCAATGACTATGTAAAACGTAAATACAGGTTCACTTAGTGTGGACAATAAGAAGTTTTGGGCTACCGTAGAGTCCTCGGAGCATTCCTTCGAGGTTCCAATCTACGCTGAGACCCTAGACGAAGCTCTGGAGTTAGCCGAATGGCAATACGTTCCGGCTGGCTTTGAGGTTACTCGTGTGCGTCCTTGTGTAGCACCGAAGTAATAc                                                                                                                                                  <  1:134808/301‑1 (MQ=255)`  
                                                                                                                                                            `aCTTCACATCTTGCTCAGAGT‑GGTGCCGAAAGATGTGGGAAGAGACATTCGACGATGCGTACACCAAGCTGTATGAACTTTGGAAATCGAGAGGTCAATGACTATGTCAAACGTAAATACAGGTTCACTTAGTGTGGACAATAAGAAGTTTTGGGCTACCGTAGAGTCCTCGGAGCATTCCGTCGAGGTTCCAATCTACGCTGAGACCCTAGACGAAGCTCTGGAGTTAGCCGAATGGCAATACGTTCCGGCTGGCTTTGAGGTTACTCGTGTGCGTCCTTGTGTAGCACCGAAGTAATAc                                                                                                                                                  <  1:47358/301‑1 (MQ=255)`  
                                                                                                                                                            `aCTTCAAAGCTGGCGAAGAGT‑GGGGCCGAAAGAGGTGGGAAGAGACATTAGACGATGCGTAAATCAAGCTGTATGAACTTTGGAAATCGAGAGGTCAATGACTATGTCAAACGTAAATACAGGTTCACTTAGTGTGGACAATAAGAAGTTTTGGGCTACCGTAGAGTCCTCGGAGCAGTCCTTCGAGGTTCCAATCTACGCTGAGACCATAGACGAAGCTCTGGAGTTAGCCGAATGGCAATACGTTCCGGCTGGCTTTGAGGTTACTCGTGTGCGTCCTTGTGTAGCACCGAAGTAATAc                                                                                                                                                  <  1:255816/301‑1 (MQ=255)`  
                                                                                                                                                             `cTTCACATCTTGCTCAGAGT‑GGTGCCGAAAGATGTGGGAAGAGACATTCGACGATGCGTACATCAAGCTGTATGAACTTTGGAAATCGAGAGGTCAATGACTATGTCAAACGTAAATACAGGTTCACTTAGTGTGGACAATAAGGAGTTTTGGGCTACCGTAGAGTCCTCGGAGCATTCCTTCGAGGTTCCAATCTACGCTGAGACCCTAGACGAAGCTCTGGAGTTAGCCGAATGGCAATACGTTCCGGCTGGCTTTGAGGTTACTCGTGTGCGTCCTTGCGTAGCACCGAAGTAATACg                                                                                                                                                 >  1:86941/1‑301 (MQ=255)`  
                                                                                                                                                             `cTTCACATCTTGCTCAGAGT‑GGTGCCGAAAGATGTGGGAAGAGACATTCGACGATGCGTACATCAAGCTGTATGAACTTTGGAAATCGAGAGGTCAATGACTATGTCAAACGTAAATACAGGTTCACTTAGTGTGGACAATAAGAAGTTTTGGGCTACCGTAGAGTCCTCGGAGCATTCCTTCGAGGTTCCAATCTACGCTGAGACCCTAGACGAAGCTCTGGAGTTAGCCGAATGGCAATACGTTCCGGCTGGCTTTGAGGTTACTCGTGTGCGTCCTTGTGTCGCACCGAAGTAATACg                                                                                                                                                 >  1:150634/1‑301 (MQ=255)`  
                                                                                                                                                               `tCACATCTTGCTCAGAGT‑GGTGCCGAAAGATGTGGGAAGAGACATTCGACGATGCGTACATCAAGCTGTATGAACTTTGGAAATCGAGAGGTCAATGACTATGTCAAACGTAAATACAGGTTCACTTAGTGTGGACAATAAGAAGTTTTGGGCTACCGTAGAGTCCTCGGAGCATTCCTTCGAGGTTCCAATCTACGCTGAGACCCTAGACGAAGCTCTGGAGTTAGCCGAATGGCAATACGTTCCGGCTGGCTTTGAGGTTACTCGTGTGCGTCCTTGTGTAGCACCGAAGTAATACGAc                                                                                                                                               <  1:265567/301‑1 (MQ=255)`  
                                                                                                                                                                   `aTCTTGCTCAGAGT‑GGTGCCGAAAGATGTGGGAAGAGACATTCGACGATGCGTACATCAAGCTGTATGAACTTTGGAAATCGAGAGGTCAATGACTATGTCAAACGTAAATACAGGTTCACTTAGTGTGGACAATAAGAAGTTTTGGGCTACCGTAGAGTCCTCGGAGCATTCCTTCGAGGTTCCAATCTACGCTGAGACCCTAGACGAAGCTCTGGAGTTAGCCGTATGGCAATACGTTCCGGCTGGCTTTGAGGTTACTCGTGTGCGTCCTTGTGTAGCACCGAAGTAATACGACTCAc                                                                                                                                           >  1:75218/1‑301 (MQ=255)`  
                                                                                                                                                                    `tCTTGCTCAGAGT‑GGTGCCGAAAGATGTGGGAAGAGACATTCGACGATGCGTACATCAAGCTGTATGAACTTTGGAAATCGAGAGGTCAATGACTATGTCAAACGTAAATACAGGTTCACTTAGTGTGGACAATAAGAAGTTTTGGGCTACCGTAGAGTCCTCGGAGCATTCCTTCGAGGTTCCAATCTACGCTGAGACCCTAGACGAAGCTCTGGAGTTAGCCGAATGGCAATACGTTCCGGCTGGCTTTGAGGTTACTCGTGTGCGTCCTTGTGTAGCACCGAAGTAATACGACTCACt                                                                                                                                          <  1:291144/301‑1 (MQ=255)`  
                                                                                                                                                                        `gCTCAGAGT‑GGTGCCGAAAGATGTGGGAAGAGACATTCGACGATGCGTACATCAAGCTGTATGAACTTTGGAAATCGAGAGGTCAATGACTATGTCAAACGTAAATACAGGTTCACTTAGTGTGGACAATAAGAAGTTTTGGGCTACCGTAGAGTCCTCGGAGCATTCCTTCGAGGTTCCAATCTACGCTGAGACCCTAGACGAAGCTCTGGAGTTAGCCGAATGGCAATACGTTCCGGCTGGCTTTGAGGTTACTCGTGTGCGTCCTTGTGTAGCACCGAAGTAATACGACGCACTATTa                                                                                                                                      >  1:65629/1‑301 (MQ=255)`  
                                                                                                                                                                        `gCTCAGAGT‑GGTGCCGAAAGATGTGGGAAGAGACATTCGACGATGCGTACATCAAGCTGTATGAACTTTGGAAATCGAGAGGTCAATGACTATGTCAAACGTAAATACAGGTTCACTTAGTGTGGACAATAAGAAGTTTTGGGCTACCGTAGAGTCCTCGGAGCATTCCTTCGAGGTTCCAATCTACGCTGAGACCCTAGACGAAGCTCTGGAGTTAGCCGAATGGCAATACGTTCCGGCTGGCTTTGAGGTTACTCGTGTGAGTCCTTGGGTAGCACCGAAGTAATACGACTCACTAttt                                                                                                                                      >  1:337653/1‑300 (MQ=255)`  
                                                                                                                                                                               `gt‑ggtgCCGAAAGATGTGGGAAGAGACATTCGACGATGCGTACATCAAGCTGTATGAACTTTGGAAATCGAGAGGTCAATGACTATGTCAACCGTAAATACAGGTTCACTTAGTGTGGACAATAAGAAGTTTTGGGCTACCGTAGAGTCCTCGGAGCATTCATTCGAGGTTCCAATCTACGCTGAGACCCTAGACGAAGCTCTGGAGTTAGCCGAATGGCAATACGTTCCGGCTGGCTTTGAGGTTACTCGTGTGCGTCCTTGTGTAGCACCGAAGTAATAGCACTCACTAtctgtctctt                                                                                                                               >  1:269269/1‑292 (MQ=255)`  
                                                                                                                                                                                       `ggAAAGATGTGGGAAGAGACATTCGACGATGCGTACATCAAGCTGTATGAACTTTGGAAATCGAGAGGTCAATGACTATGTCAAACGTAAATACAGGTTCACTTAGTGTGGACAATAAGAAGTTTTGGGCTACCGTAGAGTCCTCGGAGCATTCCTTCGAGGTTCCAATCTACGCTGAGACCCTAGACGAAGCTCTGGAGTTAGCCGAATGGCAATACGTTCCGGCTGGCTTTGAGGTTACTCGTGTGCGTCCTTGTGTAGCACCGAAGTAATATGACTCACTATTAGGGAAGACTCCctc                                                                                                                        <  1:331709/300‑1 (MQ=255)`  
                                                                                                                                                                                          `aaGATGTGGGAAGAGACATTCGACGATGCGTACATCAAGCTGTATGAACTTTGGAAATCGAGAGGTCAATGACTATGTCAAACGTAAATACAGGTTCACTTAGTGTGGACAATAAGAAGTTTTGGGCTACCGTAGAGTCCTCGGAGCATTCCTTCGAGGTTCCAATCTACGCTGAGACCCTAGACGAAGCTCTGGAGTTAGCCGAATGGCAATACGTTCCGGCTGGCTTTGAGGTTACTCGTGTGCGTCCTTGTGTAGCACCGAAGTAATACGACTCACTATTAGGGAAGACTCCCTCTga                                                                                                                     <  1:131517/301‑1 (MQ=255)`  
                                                                                                                                                                                           `aGATGTGGGAAGAGACATTCGACGATGCGTAGATCAAGCTGTATGAACTTTGGAAATCGAGAGGTCAATGACTATGTCAAACGTAAATACAGGTTCACTTAGTGTGGACAATAAGAAGTTTTGGGCTACCGTAGAGTCCTCGGAGCATTCCTTCGAGGTTCCAATCTACGCTGAGACCCTAGACGAAGCTCTGGAGTTAGCCGAATGGCAATACGTTCCGGCTGGCTTTGAGGTTACTCGTGTGCGTCCTTGTGTAGCACCGAAGTAATACGACTCACTATTAGGGAAGACTCCCTCTgag                                                                                                                    <  1:185499/301‑1 (MQ=255)`  
                                                                                                                                                                                                 `gggAAGAGACATTCGACGATGCGTACATCAAGCTGTATGAACTTTGGAAATCGAGAGGTCAATGACTATGGCAAACGGAAATACAGGTTCACTTAGTGTGGACAATAAGAAGTTTTGGGCTACCGTAGAGTCCTCGGAGCATTCCTTCGAGGTTCCAATCTACGCTGAGACCCTAGACGAAGCTCTGGAGTTAGCCGAATGGCAATACGTTCCGGCTGGCTTTTAGGTTACTCGTGTGCGTCCTTGTGTAGCACCGAAGTAATACGACTCACTATTAGGGACGACTCCCTCTTAGAAActa                                                                                                              >  1:157134/1‑299 (MQ=255)`  
                                                                                                                                                                                                         `actttCGACGATGCGTACATCAAGCTGTATGAACTTTGGAAATCGAGAGGTCAATGACTATGTCAAACGTAAATACAGGTTCACTTAGTGTGGACAATAAGAATTTTTGGGCTACCGTAGAGTCCTCGGAGCATTCCTTCGAGTTTCCAATCTACGCTGAGACCCTAGACGAAGCTCTGGAGTTAGCCGAATGGCACTACGTTCCGGCTGGCTTTGAGGTTACTCGTGTGCGTCCTTGTGTAGCACCGAAGTAATACGACTCACTATTAGGGAAGACTCCCTCTGAGAAACCAAACGAAAc                                                                                                      <  1:240704/298‑1 (MQ=255)`  
                                                                                                                                                                                                          `cATTCGACTATGCTTACATCAAGCTGTATGAACTTTGGAAATCGAGAGGTCAATGACTATGTCAAACGTAAATACAGGTTCACTTAGTTTGGACAATAAGAAGTTTTGGGCTACCGTAGAGTCCTCGGAGCATTCCTTCGAGGTTCCAATCTACGCTGAGACCCTAGACGAAGCTCTGGAGTTAGCCGAATGGCAATACGTTCCGGCTGGCTTTGAGGTTACTCGTGTGCGTCCTTGTGTAGCACCGAAGTAATACGACTCACTATTAGGGAAGACTCCCTCTGAGAAACCAAACGAAAcc                                                                                                     <  1:32941/301‑1 (MQ=255)`  
                                                                                                                                                                                                                    `tGCGTACATCAAGCTGTATGAACTTTGGAAATCGAGAGGTCAATGACTATGTCAAACGTAAATACAGGTTCACTTAGTGTGGACAATAAGAAGTTTTGGGCTACCGTAGAGTCCTCGGAGCATTCCTTCGAGGTTCCAATCTACGCTGAGACCCTAGACGAAGCTCTGGAGTTAGCCGAATGGCAATACGTTCCGGCTGGCTTTGAGGTTACTCGTGTGCGTCCTTGTGTAGCACCGAAGTAATACGACTCACTATTAGGGAAGACTCCCTCTGAGAAACCAAACGAAACCTAAAggctgt                                                                                           >  1:125537/1‑297 (MQ=255)`  
                                                                                                                                                                                                                     `gCGTACATCAAGCTGTATGAACTTTGGAAATCGAGAGGTCAATGACTATGTCAAACGTAAATACAGGTTCACTTAGTGTGGACAATAAGAAGTTTTGGGCTACCGTAGAGTCCTCGGAGCATTCCTTCGAGGTTCCAATCTACGCTGAGACCCTAGACGAAGCTCTGGAGTTAGCCGAATGGCAATACGTTCCGGCTGGCTTTGAGGTTACTCGTGTGCGTCCTTGTGTAGCACCGAAGTAATACGACTCACTATTAGGGAAGACTCCCTCTGAGAAACCAAACGAAACCTAAAGGAGAtt                                                                                          >  1:122467/1‑301 (MQ=255)`  
                                                                                                                                                                                                                     `gCGTACATCAAGCTGTATGAACTTTGGAAATCGAGAGGTCAATGACTATGTCAAACGTAAATACAGGTTCACTTAGTGTGGACAATAAGAAGTTTTGGGCTACCGTAGAGTCCTCGGAGCATTCCTTCGAGGTTCCAATCTACGCTGAGACCCTAGACGAAGCTCTGGAGTTAGCCGAATGGCAATACGTTCCGGCTGGCTTTGAGGTTACTCGTGTGCGTCCTTGTGTAGCACCGAAGTAATACGACTCACTATTAGGGAAGACTCCCTCTGAGAAACCAAACGAAACCTAAAGGAGAtt                                                                                          >  1:326958/1‑301 (MQ=255)`  
                                                                                                                                                                                                                         `aCATCAAGCTGTATGACCTTTGGAAATCGAGAGGTCAATGACTATGTCAAACGTAAATACAGGTTCACTTAGTGTGGACAATACGAAGTTTTGGGCTACCGTAGAGTCCTCTGAGCATTCCTTCGAGGTTCCAATCTACGCTGAGACCCTAGACGAAGCTCTGGAGTTAGCCGAATGGCAATACGTTCCGGCTGGCTTTGAGGTTACTCGTGTGCGTCCTTGTGTAGCACCGAAGTAATACGACCTACTATTAGGGAAGACTCCCTCTGAGAAACCAACCGAAACCTAAAGGCGATTAAcc                                                                                      >  1:230428/1‑300 (MQ=255)`  
                                                                                                                                                                                                                         `aCATCAAGCTGTATGAACTTTGGAAATCGAGAGGTCAATGACTATGTCAAACGTAAATACAGGTTCACTTAGTGTGGACAATAAGAAGTTTTGGGCTACCGTAGAGTCCTCGGAGCATTCCTTCGAGGTTCCAATCAACGCTGAGACCCTAGACGAAGCTCTGGAGTTAGCCGAATGGCAATACGTTCCGGCTGGCTTTGAGGTTACTCGTGTGCGTCCTTGTGTAGCACCGAAGTAATACGACTCACGATTAGGGAAGACTCACTCTGAGAAACCAAACGAAACCTAAAGGAGATTAACa                                                                                      >  1:33503/1‑301 (MQ=255)`  
                                                                                                                                                                                                                          `ctcggagatgtgtataagagacagaTCGAGAGGTCAATGACTATGTCAAACGTAAATACAGGTTCACTTAGTGTGGACAATAAGAAGTTTTGGGCTACCGTAGAGTCCTCGGAGCATTCCTTCGAGGTTCCAATCTACGCTGAGACCCTAGACGAAGCTCTGGAGTTAGCCGAATGGCAATACGTTCCGGCTGGCTTTGAGGTTACTCGTGTGCGTCCTTGTGTAGCACCGAAGTAATACGACTCACTATTAGGGAAGACTCCCTCTGAGAAACCAAACGAAACCTAAAGGAGATTAACAt                                                                                     <  1:3908/277‑1 (MQ=255)`  
                                                                                                                                                                                                                          `cagcAAGCTGTATGAACTTTGGAAATCGAGAGGTCAATGACTATGTAAAACGTAAATACAGGTTCACTTAGTGAGGACAATAAGAAGTTTTGGGCTACCGTAGAGTCCTCGGAGCATTCCTTCGAGGTTCCAATCTACGCTGAGACCCTAGACGAAGCTCTGGAGTTAGCCGAATGGCAATACGTTCCGGCTGGCTTTGAGGTTACTCGTGTGCGTCCTTGTGTAGCACCGAAGTAATACGACTCACTATTAGGGAAGACTCCCTCTGAGAAACCAAACGAAACCTAAAGGAGATTAACAt                                                                                     <  1:110335/298‑1 (MQ=255)`  
                                                                                                                                                                                                                          `cATCAAGCTGTATGAACTTTGGAAATCGAGAGGTCAATGACTATGTCAAACGTAAATACAGGTTCACTTAGTGTGGACAATAAGAAGTTTTGGGCTACCGTAGAGTCCTCGGAGCATTCCTTCGAGGTTCCAATCTACGCTGAGACCCTAGACGAAGCTCTGGAGTTAGCCGAATGGCAATACGTTCCGGCTGGCTTTGAGGTTACTCGTGTGCGTCCTTGTGTAGCACCGAAGTAATACGACTCACTATTAGGGAAGACTCCCTCTGAGAAACCAAACGAAACCTAAAGGAGATTAACAt                                                                                     <  1:120427/301‑1 (MQ=255)`  
                                                                                                                                                                                                                          `cATCAAGCTGTATGAACTTTGGAAATCGAGAGGTCAATGACTATGTCAAACGTAAATACAGGTTCACTTAGTGTGGACAATAAGAAGTTTTGGGCTACCGTAGAGTCCTCGGAGCATTCCTTCGAGGTTCCAATCTACGCTGAGACCCTAGACGAAGCTCTGGAGTTAGCCGAATGGCAATACGTTCCGGCTGGCTTTGAGGTTACTCGTGTGCGTCCTTGTGTAGCACCGAAGTAATACGACTCACTATTAGGGAAGACTCCCTCTGAGAAACCAAACGAAACCTAAAGGAGATTAACAt                                                                                     <  1:112868/301‑1 (MQ=255)`  
                                                                                                                                                                                                                                `gCTGTATGAACTTTGGAAATCGAGAGGTCAATGACTATGTCAAACGTAAATACAGGTTCACTTAGTGTGGACAATAAGAAGTTTTGGGCTACCGTAGAGTCCTCGGAGCATTCCTTCGAGGTTCCAATCTACGCTGAGACCCTAGACGAAGCTCTGGAGTTAGCCGAATGGCAATACGTTCCGGCTGGCTTTGAGGTTACTCGTGTGCGTCCTTGTGTAGCACCGAAGTAATACGACTCACTATTAGGGAAGACTCCCTCTGAGAAACCAAACGAAACCTAAAGGAGATTAACATTATGGc                                                                               <  1:128857/301‑1 (MQ=255)`  
                                                                                                                                                                                                                                   `gTATGAACTTTGGAAATCGAGAGGTCAATGACTATGTCAAACGTAAATACAGGTTCACTTAGTGTGGACAATAAGAAGTTTTGGGCTACCGTAGAGTCCTCGGAGCATTCCTTCGAGGTTCCAATCTACGCTGAGACCCTAGACGAAGCTCTGGAGTTAGCCGAATGGCAATACGTTCCGGCTGGCTTTGAGGTTACTCTTGTGCGTCCTTGTGTATCACCGAAGCAATACGACTCACTATTAGGGAAGACTCCCTCTGAGAAACCAAACGACACCTAACGCAGCTTAACATTTTggttta                                                                            >  1:299795/1‑297 (MQ=255)`  
                                                                                                                                                                                                                                   `gTATGAACTTTGGAAATCGAGAGGTCAATGACTATGTCAAACGTAAATACAGGTTAACTTAGTGTGGACAATAAGAAGTTTTGGGCTACCGTAGAGTCCTCGGAGCATTCCTTCGAGGTTCCAATCTACGCTGAGACACTAGACGAAGCTCTGGAGTTAGCCGAATGGCAATACGTTCCGGCTGGCTTTGAGGTTACTCGTGTGCGTCCTTGTGTAGCACCGAAGTAATACGACTCACTATTAGGGAAGACTCCCTCTGAGAAACCAAACGAAACCTAAAGGAGATTAACATTATGGcctg                                                                            >  1:180475/1‑298 (MQ=255)`  
                                                                                                                                                                                                                                     `aTGAACTTTGGAAATCGAGAGGTCAATGACTATGTCAAACGTAAATACAGGTTCACTTAGTGTGGACAATAAGAAGTTTTGGGCTACCGTAGAGTCCTCGGAGCATTCCTTCGAGGTTCCAATCTACGCTGAGACCCTAGACGAAGCTTTGGAGTTAGCCGAATGGCAATACGTTCCGGCTGGCTTTGAGGTTACTCGTGTGCGTCCTTGTGTAGCACCGAAGTAATACGACTCACTATTAGGGAAGACTCCCTCTGAGAAACCAAACGAAACCTAAAGGAGATTAACATTATGGCTaaga                                                                          <  1:313237/301‑1 (MQ=255)`  
                                                                                                                                                                                                                                       `gAACTTTGGAAATCGAGAGGTCAATGACTATGTCAAACGTAAATACAGGTTCACTTAGTGTGGACAATAAGAAGTTTTGGGCTACCGTAGAGTCCTCGGAGCATTCCTTCGAGGTTCCAATCTACGCTGAGACCCTAGACGAAGCTCTGGAGTTAGCCGAATGGCAATACGTTCCGGCTGGCTTTGAGGTTACTCGTGTGCGTCCTTGTGTAGCACCGAAGTAATACGACTCACTATTAGTGAAGACTCCCTCTGAGAAACCAAACGAAACCTAACGGAGATTAACATTATGGCTaagaaa                                                                        >  1:21084/1‑300 (MQ=255)`  
                                                                                                                                                                                                                                                          `gTCAATGACTATGTCAAACGTAAATACAGGTTCACTTAGTGTGGACAATAAGAAGTTTTGGGCTACCGTAGAGTCCTCGGAGCATTCCTTCGAGGTTCCAATCTACGCTGAGACCCTAGACGAAGCTCTGGAGTTAGCCGAATGGCAATACGTTCCGGCTGGCTTTGAGGTTACTCGTGTGCGCCCTTGTGTAGCACCGAAGTAATACGACTCACTATTAGGGAAGACTCCCTCTGAGAAACCAAACGAAACCTAAAGGAGATTAACATTATGGcctgtctcttatacacatctccgagcc                                                     >  1:191227/1‑275 (MQ=255)`  
                                                                                                                                                                                                                                                            `cAATGACTATGTCAAACGTAAATACAGGTTCACTTAGTGTGGACAATAAGAAGTTTTGGGCTACCGTAGAGTCCTCGGAGCATTCCTTCGAGGTTCCAATCTACGCTGAGACCCTAGACGAAGCTCTGGAGTTAGCCGAATGGCAATACGTTCCGGCTGGCTTTGAGGTTACTCGTGTGCGTCCTTGTGTAGCACCGAAGTAATACGACTCACTATTAGGGAAGACTCCCTCTGAGAAACCAAACGAAACCTAAAGGAGATTAACATTATGGCTAAGAAGATTTTCACCTCTGCGCTGGGt                                                   <  1:199795/301‑1 (MQ=255)`  
                                                                                                                                                                                                                                                              `cagACTATTTCAAACGTAAATACAGGTTCACTTAGTGTGGACAATAAGAATTTTTGGTCTACCTTAGAGTCCTCGGAGCATTCCTTCGAGTTTCCAATCTACGCTGAGACCCTAGACGAAGCTCTGGAGTTAGCCGAATGGCAATACGTTCCGTCTGGCTTTGAGGTTACTCGTGTGCGTCCTTGTGTAGCACCGAAGTAATACGACTCACTATTAGGGAATACTCCCTCTGAGAAACCAAACGAAACCTAAAGGAGATTAACATTATTGCTAAGAAGATTTTCACCTCTGCGCTGGGTAc                                                 <  1:56625/299‑1 (MQ=255)`  
                                                                                                                                                                                                                                                                      `ggtgtataagggacagggTTCACTTAGTGTGGACAATAAGAAGTTTTGGGCTAGCGTAGAGTCCTCGGAGCATTCCTTCGAGGTTCCAATCTACGCTGAGACCCTAGACGAAGCTCTGGAGTTAGCAGAATGGCAATACGTTCCGGCTGGCTTTGAGGTTACTCGTGTGCGTCCTTGTGTAGCACCGAAGTAATACGACACACTATTAGGGAAGACTCCCTCTGAGAAACCAAACGAAACCTAAAGGAGATTAACATTATGGCTAAGAAGATTTTCACCTCTGCGCTGGGTACCGCTGAAc                                         <  1:53188/285‑1 (MQ=255)`  
                                                                                                                                                                                                                                                                       `tCAAACGTAAATACAGTTTCACTTAGTGTGGATAATAAGAAGTTTTTGGCTACCGTAGAGTCCTCGGAGCATTCCTTCGAGGTTCCAATCTACGCTGAGACCCTAGACGAAGCTCTGGAGTTAGCCGAACGGCAAGACGTTCCGGCTGGCTTTGAGGTTACTCGTGTGCGTCCTTGTGTAGCACCGAAGTAATACGACTCACTATGAGGGAAGACTCCCTCTGAGAAAACAAACGAAACCTAAAGGAGATTAACATTATGGCTAAGAAGAGTTTCACCTCTGCGCTGGGTACCGCTGAAcc                                        <  1:288201/301‑1 (MQ=255)`  
                                                                                                                                                                                                                                                                           `aCGTAAATACAGGTTCACTTAGTGTGGACAATAAGAAGTTTTGGGCTACCGTAGAGTCCTCGGAGCATTCCTTCGAGGTTCCAATCTACGCTGAGACCCTAGACGAAGCTCTGGAGTTAGCCGAATGGCAATACGTTCCGGCTGGCTTTGAGGTTACTCGTGTGCGTCCTTGTGTAGCACCGAAGTAATACGACTCACTATTAGGGAAGACTCCCTCTGAGAAACCAAACGAAACCTAAAGGAGATTAACATTATGGCTAAGAAGATTTTCACCTCTGCGCTGGGTACCGCTGAACCTTAc                                    >  1:88859/1‑301 (MQ=255)`  
                                                                                                                                                                                                                                                                             `gTAAATACAGGTTAACTTAGTGTGGACAATATGAAGTTTTGGGCTACCGTAGAGTCCTCGGAGCATTCCTTCGAGGTTCCAATCTACGCTGAGACCCTAGACGAAGCTCTGGAGTTAGCCGAATGGCAATACGTTCCGGCTGGCTTTGAGGTTACTCGTGTGCGTCCTTGTGTAGCACCGAAGTAATACGACTCACTATTAGGGAAGACTCCCTCTGAGAAACCAAACGAAACCTAAAGGAGATTAACATTATGGCTAAGAAGATTTTCACCTCTGCGCTGGGTACCGCTGAACCTTACGc                                  <  1:100052/301‑1 (MQ=255)`  
                                                                                                                                                                                                                                                                                   `gtgtataagagacaggtgGACAATAAGAAGTTTTGGGCTACCGTAGAGTCCTCGGAGCATTCCTTCGAGGTTCCAATCTACGCTGAGACCCTAGACGAAGCTCTGGAGTTAGCCGAATGGCAATACGTTCCGGCTGGCTTTGAGGTTACTCGTGTGCGTCCTTGTGTAGCACCGAAGTAATACGACTCACTATTAGGGAAGACCCCCTCTGAGAAACCAAACGAAACCTAAAGGAGATTAACATTATGGCTAAGAAGATTTTCACCTCTGCGCTGGGTACCGCTGAACCTTACGCTTACAt                            <  1:321152/286‑1 (MQ=255)`  
                                                                                                                                                                                                                                                                                    `cAGGTTCACTTAGTGTGGACAATAAGAAGTTTTGGGCTACCGTAGAGTCCTCGGAGCATTCCTTCGAGGTTCCAATCTACGCTGAGACCCTAGACGAAGCTCTGGAGTTAGCCGAATGGCAATACGTTCCGGCTGGCTTTGAGGTTACTCGTGTGCGTCCTTGTGTAGCACCGAAGTAATACGACTCACTATTAGGGAAGACTCCCTCTGAGAAACCAAACGAAACCTAAAGGAGATTAACATTATGGCTAAGAAGATTTTCACCTCTGCGCTGGGTACCGcctgtctcttatacacatct                           >  1:139643/1‑282 (MQ=255)`  
                                                                                                                                                                                                                                                                                              `tAGTGTGGACAATAAGAAGTTTTGGGCTACCGTAGAGTCCTCGGAGCATTCCTTCGAGGTTCCAATCTACGCTGAGACCCTAGACGAAGCTCTGGAGTTAGCCGAATGGCAATACGTTCCGGCTGGCTTTGAGGTTACTCGTGTGCGTCCTTGTGTAGCACCGAAGTAATACGACTCACTATTAGGGAAGACTCCCTCTGAGAAACCAAACGAAACCTAAAGGAGATTAACATTATGGCTAAGAAGATTTTCACCTCTGCGCTGGGTACCGCTGAACCTTACGCTTACATCGCCAAGCCgg                 >  1:258076/1‑301 (MQ=255)`  
                                                                                                                                                                                                                                                                                                       `cAATAAGACGTTTTGGGCTACCGTAGAGTCCTCGGAGCATTCCTTCGAGGTTCCAATCTACGCTGAGACCCTAGACGAAGCTCTGGAGTTAGCCGAATGGCAATACGTTCCGGCTGGCTTTGAGGTTACTCGTGTGCGTCCTTGTGTAGCACCGAAGTAATACGACTCACTATTAGGGAAGACTCCCTCTGAGAAACCAAACGAAACCTAAAGGAGATTAACATTATGGCTAAGAAGATTTTCACCTCTGCGCTGGGTACCGCTGAACCTTACGCTTACATCGCCAAGCCGGACTACGGCa        >  1:233729/1‑301 (MQ=255)`  
                                                                                                                                                                                                                                                                                                         `aTAAGAAGTTTTGGGCTACCGTAGAGTCCTCGGAGCATTCCTTCGAGGTTCCAATCTACGCTGAGACCCTAGACGAAGCTCTGGAGTTAGCCGAATGGCAATACGTTCCGGCTGGCTTTGAGGTTACTCGTGTGCGTCCTTGTGTAGCACCGAAGTAATACGACTCACTATTAGGGAAGACTCCCTCTGAGAAACCAAACGAAACCTAAAGGAGATTAACATTATGGCTAAGAAGATTTTCACCTCTGCGCTGGGTACCGCTGAACCTTACGCTTACATCGCCAAGCCGGACTACGGCAAc      <  1:34007/301‑1 (MQ=255)`  
                                                                                                                                                                                                                                                                                                           `aagaagTTTTGGGCTACCGTAGATTCATCGGAGCATTCCTTTGAGGTTCCAATCTACGGTGAGACCCTAGAGGAAGCTCTGGAGTTAGCCGAATGGCAATACGTTCCGGCTAGCTTTGAGGTTACTCGTGTGCGTCCTTGGGTAGCACCGAAGTAATACGACTCACTATTAGGGAAGACTCCCTCTGAGAAACCAAACGAAACCTAAAGGAGATTAACATTATGACTAAGAAGATTTTCACCTCTGCGCTGGGTACCGCTGAACCTTACGCTTACATCGCCAAGCCGGACTACGGCAACGa    <  1:159679/301‑1 (MQ=255)`  
                                                                                                                                                                                                                                                                                                             `gaagTTTTGGGCTACCGTAGAGTCCTCGGAGCATTCCTTCGAGGTTCCAATCTACGCTGAGACCCTAGAAGAAGCTCTGGAGTTAGCCGAATGGCAATACGTTCCGGCTGGCTTTGAGGTTACTCGTGTGCGTCCTTGTGTAGCACCGAAGTAATACGACTCACTATTAGGGAAGACTCCCTCTGAGAAACCAAACGAAACCTAAAGGAGATTAACATTATGGCTAAGAAGATTTTCACCTCTGCGCTGGGTACCGCTGAACCTTACGCTTACATCGCCAAGCCGGACTACGGCAACGAag  <  1:168418/301‑1 (MQ=255)`  

                                                                                                                                                                                                                                                                                                             `|`                                                                                                                                                                                                                                                                                                              
`AAGTACAGACTACGTGCTGGTAAGAAGTCAGAGTTAGCGTACTTAGAGAAAGACCTAGCGAAAGCAGACTTCTATAAAGAACTCTTTGAGAAACATAAGGATAAATGTTATGCATAACTTCAAGTCAA‑CCCCACCTGCCGACAGCCTATCTGATGACTTCACATCTTGCTCAGAGT‑GGTGCCGAAAGATGTGGGAAGAGACATTCGACGATGCGTACATCAAGCTGTATGAACTTTGGAAATCGAGAGGTCAATGACTATGTCAAACGTAAATACAGGTTCACTTAGTGTGGACAATAAGAAGTTTTGGGCTACCGTAGAGTCCTCGGAGCATTCCTTCGAGGTTCCAATCTACGCTGAGACCCTAGACGAAGCTCTGGAGTTAGCCGAATGGCAATACGTTCCGGCTGGCTTTGAGGTTACTCGTGTGCGTCCTTGTGTAGCACCGAAGTAATACGACTCACTATTAGGGAAGACTCCCTCTGAGAAACCAAACGAAACCTAAAGGAGATTAACATTATGGCTAAGAAGATTTTCACCTCTGCGCTGGGTACCGCTGAACCTTACGCTTACATCGCCAAGCCGGACTACGGCAACGAAG  >  NC_001604.1/8640‑9239`

  

Alignment Legend

Aligned base mismatch/match (shaded by quality score): `ATCG/ATCG` `< 3 ≤` `ATCG/ATCG` `< 11 ≤` `ATCG/ATCG` `< 24 ≤` `ATCG/ATCG` `< 35 ≤` `ATCG/ATCG` `< 38 ≤` `ATCG/ATCG`

Unaligned base: `atcg`    Masked matching base: `atcg`    Alignment gap: `‑`    Deleted base: `‑`

Reads not counted as support for junction

read\_name Not counted due to insufficient overlap past the breakpoint.

read\_name Not counted due to not crossing MOB target site duplication.
